# MicroRNA-17-5p regulates EMT by targeting vimentin in colorectal cancer

**DOI:** 10.1038/s41416-020-0940-5

**Published:** 2020-06-17

**Authors:** Tae Won Kim, Yeo Song Lee, Nak Hyeon Yun, Chang Hoon Shin, Hye Kyung Hong, Hyeon Ho Kim, Yong Beom Cho

**Affiliations:** 1 Department of Health Sciences and Technology, SAIHST, Sungkyunkwan University, Seoul, Republic of Korea; 2 Sungkyunkwan University School of Medicine, Seoul, Republic of Korea; 3 Institute for Future Medicine, Samsung Medical Center, Seoul, Republic of Korea; 4 Department of Surgery, Samsung Medical Center, Sungkyunkwan University School of Medicine, Seoul, Republic of Korea

**Keywords:** Metastasis, Colorectal cancer

## Abstract

**Background:**

Epithelial–mesenchymal transition (EMT) is the most common cause of death in colorectal cancer (CRC). In this study, we investigated the functional roles of miRNA-17-5p in EMT of CRC cells.

**Methods:**

In order to determine if miRNA-17-5p regulated EMT, the precursors and inhibitors of miR-17-5p were transduced into four CRC cells. To evaluate the regulatory mechanism, we performed argonaute 2 (Ago2) immunoprecipitation (IP) and luciferase assay. In addition, we used an intra-splenic injection mouse model of BALB/c nude mice to investigate the metastatic potential of miRNA-17-5p in vivo.

**Results:**

The miRNA-17-5p expression was lower in primary CRC tissues with metastasis than in primary CRC tissues without metastasis in our RNA sequencing data of patient tissue. Real-time quantitative PCR revealed that miRNA-17-5p was inversely correlated with that of vimentin in five CRC cell lines. Over-expression of miRNA-17-5p decreased vimentin expression and inhibited cell migration and invasion in both LoVo and HT29 cells. However, inhibition of miRNA-17-5p showed the opposite effect. Ago2 IP and luciferase assay revealed that miRNA-17-5p directly bound to the 3′UTR of *VIM* mRNA. Furthermore, miRNA-17-5p inhibited the metastasis of CRC into liver in vivo.

**Conclusions:**

Our results demonstrated that miRNA-17-5p regulates vimentin expression, thereby regulating metastasis of CRC.

## Background

Colorectal cancer (CRC) is one of the most commonly diagnosed cancers worldwide.^1–3^ It is also a major health threat and an important cause of cancer-related mortality.^1^ However, studies of specific and appropriate drugs for CRC have not reported successful outcomes.^4^ CRC metastasis is the main cause of the high mortality and poor outcome.^4^ During several steps of metastasis, the cancer cells disseminate into other organs via epithelial−mesenchymal transition (EMT). Hence, many studies have proposed EMT as a mechanism of metastasis in CRC.^5,6^ However, the molecular mechanisms underlying EMT in CRC remain poorly understood.

MicroRNAs (miRNAs) are small non-coding RNAs containing short single strands (~25 nucleotides in length) and are known to regulate the gene expression by binding to the 3′-untranslated region (3′UTR) of the target mRNAs.^6,7^ It has been reported that the dysregulation of miRNAs is closely associated with the initiation and development of cancer, including metastasis.^6,8–10^ MiRNAs act as oncogenes or tumour suppressors according to their target genes.^11^ Additionally, miRNAs using non-invasive samples yield enhanced the sensitivity and specificity compared with the currently used CRC screening methods based on genomics, methylomics or proteomics.^11^ To identify the metastasis-associated miRNAs in CRC, we obtained RNA and miRNA sequencing data from 43 CRC patients treated in the Samsung Medical Center. Analysis of the data revealed that miRNA-17-5p (miR-17-5p) was related to metastasis. In addition, we reviewed studies investigating EMT-related miR-17-5p. However, miR-17-5p yielded controversial results.

It is well known that the functional roles of miRNA are mainly dependent on the cell context. Therefore, the same miRNA may yield different results depending on the cell type or condition. Each miRNA containing similar seed sequences has different functions in the specific cancer type.^12^ For example, miR-17-5p promotes EMT in osteosarcoma and retinoblastoma,^13,14^ whereas it inhibits EMT in non-small-cell lung cancer and breast cancer.^15,16^ The differences between promotion and inhibition of miRNA-related EMT are attributed to the organ-specific function of miRNA. The function of miRNA in colon cancer has been strongly disputed. Xi et al.^17^ reported that miR-17-5p induces EMT by regulating CYP7B1 expression. However, conflicting results were reported by another group suggesting that miR-17-5p cluster reduced EMT in a CRC orthotopic mouse model.^18^ Therefore, we have resolved the disputed results via both in vitro and in vivo validation.

The purpose of our study was to elucidate the function of miR-17-5p in CRC metastasis and to investigate the mechanism underlying miR-17-5p-related EMT in various CRC cells.

## Methods

### Cell culture and transfection

Colorectal cancer cell lines (HCT15, Colo205, LoVo, HT29, SW480, HCT116, DLD-1, SW620, SW48, LS513, and RKO) were purchased from American Type Culture Collection (ATCC) and cultured with RPMI 1640 (Gibco, Grand Island, NY, USA) supplemented with 10% foetal bovine serum (FBS, Gibco) and 1% penicillin-streptomycin (Gibco) in a 37 °C incubator with 5% CO_2_. For transient transfection, cells were transfected with indicated siRNA or miRNA using Lipofectamine RNAiMAX (Invitrogen, Carlsbad, CA, USA) according to the manufacturer’s protocol. SiRNAs and miRNAs used in this study were as follows: control miRNA (4464058, Applied Biosystems), and vimentin siRNA (sc-29522, Santa Cruz Biotechnology, CA, USA), miR-17-5p mimic (4464066, Applied Biosystems), and miR-17-5p inhibitor (4464084, Applied Biosystems). All experiments were performed with mycoplasma-free cells.

### Cell lysis and western blot analysis

To prepare whole-cell extract, cells were lysed using Pro-prep buffer (Intron Biotechnology, Seoul, Korea) including protease inhibitors. Similar amounts of protein extracts were resolved via SDS-PAGE and transferred to polyvinylidene fluoride membranes. The membranes were probed with primary antibodies followed by incubation with secondary antibodies conjugated to horseradish peroxidase (Santa Cruz Biotechnology, CA, USA). β-actin was used as a loading control in western blot analysis. Antibodies used for this study were as follows: E-cadherin (24E10) (#3195, Cell Signaling Technology, Danvers, USA), vimentin (#MA5-11883, Thermo Fisher Scientific, MA, USA), and β-actin (#3700, Cell Signaling Technology).

### Cell migration and invasion assays

Cell lines were seeded onto six-well plates. When the cell confluence reached about 80% and above at around 48 h post transfection, scratch wounds were made by scraping the cell layer across each culture plate using the tip of a 10 μl pipette. After wounding, the debris was removed by washing the cells with phosphate buffered saline (PBS). Wounded cultures were incubated in serum-free medium for 30 h, and then three fields (10×) were randomly picked from each scratch wound and visualised by microscopy to assess cell migration ability. The experiments were performed in triplicate.

Cell invasion assays were carried out using a Matrigel invasion chamber (Corning, USA) hydrated for at least 2 h with 500 μl serum-free RPMI in the bottom of the well and 500 μl in the top of the chamber. After hydration of the Matrigel, RPMI in the bottom of the well was replaced with 700 μl of RPMI containing 10% FBS. Cell lines (5 × 10^4^/well) were loaded in invasion chambers with 500 μl of serum-free RPMI medium. In the lower chambers, 700 μl of RPMI supplemented with 10% FBS was added as a chemo-attractant. Plates were incubated for 24 or 48 h and then stained with haematoxylin and eosin.

### Real-time quantitative polymerase chain reaction (RT-qPCR) analysis

Total RNA was isolated using TRIzol reagent (Invitrogen) according to the manufacturer’s instructions and used as a template to synthesise complementary DNA (cDNA) using Accupower cyclescript RT premix dT20 (#K-2044-B, Bioneer, Daejeon, Korea). The levels of mRNA were quantified by RT-qPCR (ABI Prism 7900) using power SYBR^®^ Green PCR Master Mix (Applied Biosystems). In case of miR-17-5p, miRNA-specific TaqMan primer (#4427975, ID: 000393, Applied Biosystems) was used for RT-qPCR. *VIM* primer (#1: F: CCCTCACCTGTGAAGTGGAT R: GCTTCAACGGCAAAGTTCTC, #2: F: CGAAAACACCCTGCAATCTT R: ATTCCACTTTGCGTTCAAGG) and GAPDH primer (F: TGCACCACCAACTGCTTAGC R: GGCATGGACTGTGGTCATGAG) were used for RT-qPCR.

### Ribonucleoprotein immunoprecipitation (RNP-IP)

For RNP-IP, the Dynabeads^®^ Protein G (Thermo Fisher Scientific, Rockford, IL, USA) were coated with control IgG (Santa Cruz Biotechnologies, Santa Cruz, CA, USA) or Ago2 antibody (Sigma, St. Louis, MO, USA). Cytoplasmic lysates were prepared using the protein extraction buffer (PEB) containing protease/phosphatase inhibitors and RNaseOUT (Invitrogen, Carlsbad, CA, USA). Equal amounts of lysates were incubated with antibody-coated Dynabeads for 4 h at 4 °C. After washing several times with PEB buffer, the Ago2-IP materials were treated with DNase I (Ambion, Austin, TX, USA) and protease K (Bioneer, Daejeon, South Korea). Proteins were denatured with acid phenol (Ambion) and RNAs were precipitated with absolute ethanol overnight at −20 °C. The level of mRNA in Ago2-mediated RNA-induced silencing complex (RISC) was determined by RT-qPCR as described above.^19^

### Luciferase assay

To determine the miRNA recognition elements in the 3′UTR of *VIM* mRNA, we prepared four luciferase reporter constructs as follows: (A) blank pmirGLO vector, (B) wild-type 3′UTR of vimentin mRNA (GTTTCCCCTAAACCGCTAGGAGC), (C) mutant 3′UTR of vimentin mRNA in the miR-17-5p seed region (CCCTTTGCTAAACCGCTAGGAGC), and (D) perfect match in the miR-17-5p seed region (GTTTCACCTAAACCGCTAGGAGC). Luciferase activities were measured using a luminometer according to the manufacturer’s instructions (Glomax20/20 luminometer, Promega) 24 h post transfection using the Dual-Glo luciferase activity assay system (Promega). Renilla luciferase activity was normalised using firefly luciferase activity for each sample.

### Intra-splenic injections

Six- to seven-week-old female BALB/c nude mice (Provided Orient-Bio in Korea) were anesthetised with a mixture of Catamin (#7001, Yuhan) (30 mg/kg) and Rompun (Bayer) (10 mg/kg) via intra-peritoneal injection (0.01 ml/mg). A small left abdominal flank incision was made, and the spleen was exteriorised for intra-splenic injection. LoVo cells with NC-mimic (Control group, 1.2 × 10^6^ cells) and LoVo cells with miR-17-5p mimic (Experimental group, 1.2 × 10^6^ cells) were suspended in 50 μl Hanks’ balanced salt solution (HBSS) (Gibco) and injected into a mouse spleen with a 30-gauge needle. To prevent tumour cell leakage and bleeding, a cotton swab was held over the site of injection for 1 min. The injected spleen was returned to the abdomen and the wound was sutured with 6-0 black silk.

When we conducted the sacrifice, we placed the mice in the chamber and introduced 100% carbon dioxide. After we observed each mouse for lack of respiration and faded eye colour, we maintained the carbon dioxide flow for a minimum of 1 min after respiration ceases.

When female BALB/c nude mice were 6- to 7-week-old, we carried out intra-splenic injection. After 6 weeks, we checked the MRI image and sacrificed the mice for obtaining tissue.

The animal experiments were tested in specific pathogen-free animal experiments centre at the Samsung Medical Center.

To establish whether miR-17-5p activity was related to metastasis in vivo, we developed a metastatic mouse model. The intra-splenic injection model displayed the fastest approach to develop a liver metastasis mouse model than caecal-injection mouse model. According to our previous research, these anaesthesia methods can minimise the side effects and death^20^, and we also used local anaesthetics after surgery to further reduce the pain.

Ethics approval for animal use was obtained from the Samsung Medical Center on Laboratory Animals Committee (approval number: 20180129002). We carried out animal experiments in accordance with the ARRIVE reporting guidelines (attached supplementary file).

### Human specimens

Primary tumour tissues were obtained from 47 CRC patients in Samsung Medical Center and prepared for miRNA and mRNA expression profiling. Among the 47 patients, 14 of them did not have metastasis while the other 33 patients had liver metastasis. Both small RNA-seq and whole transcriptome-sequencing were conducted for these 47 primary tumour samples, where expression levels of 2669 miRNAs and 23,956 genes were acquired.

### Statistical analysis

Statistical comparisons were performed using GraphPad Prism. The data are expressed as mean ± standard deviation (SD). *P* values < 0.05 were considered statistically significant (*P < 0.05, **P < 0.01, or ***P < 0.001).

## Results

### miR-17-5p expression is inversely correlated with the expression of vimentin in colon cancer cells

**Fig. 1:**
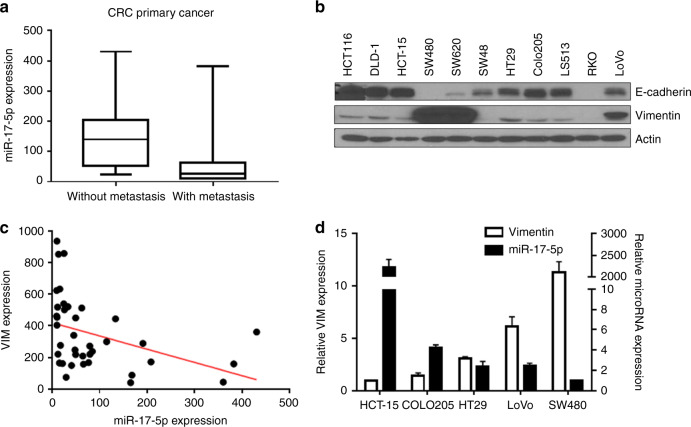
miR-17-5p is inversely correlated with the expression of vimentin in colon cancer cells. **a** miR-17-5p expression was lower in primary CRC tissues with metastasis compared with primary CRC tissues without metastasis (*p* = 0.002). **b** The cells were harvested and subsequently analysed for the differential expression of proteins by western blot. β-actin was used as the loading control. E-cadherin and vimentin were used in western blotting analysis. **c** The expression of vimentin was negatively correlated with that of miR-17-5p in clinical specimens. **d** The expression of miR-17-5p and vimentin was examined in CRC cell lines by qRT-PCR. The miR-17-5p was inversely correlated with vimentin expression.

In order to investigate the expression of specific miRNAs of CRC, we obtained RNA and miRNA sequencing data from 43 CRC patients. We found that 29 CRC patients showed liver metastasis and 14 patients did not. Analysis of RNA sequencing data of patient tissue indicated that the miR-17-5p expression was lower in primary CRC tissues with metastasis than in primary CRC tissues without metastasis (Fig. 1a). To determine if miR-17-5p was involved in the EMT process, the levels of E-cadherin and vimentin were analysed by western blot in 11 colon cancer cells (Fig. 1b). Spearman correlation analysis of our sequencing data suggested a negative correlation between miR-17-5p and vimentin expression (Fig. 1c). In addition, Spearman correlation analysis of TCGA data from cBIOPortal^21,22^ (https://www.cbioportal.org/, TCGA, PanCancer Atlas) showed a negative correlation between miR-17-5p and *VIM* expression (Supplementary Fig. S1A, *R* = −0.4591, *P* < 0.0001). Furthermore, disease-free survival analysis of TCGA data indicated that the low-level group of miR-17-5p showed poor prognosis compared to the high-level group (Supplementary Fig. S1B, *P* = 0.0189) and the high-level group of *VIM* expression showed poor prognosis than the low-level group (Supplementary Fig. S1C, *P* = 0.0386). The high group of each gene is the top 30% of all patients in gene expression, and the low group is the bottom 30% of all patients. Based on the expression of vimentin, five colon cancer cells were selected for further studies: (1) low-expressing group: HCT-15 and Colo205; (2) medium-expressing group: HT29 and LoVo; and (3) high-expressing group: SW480. RT-qPCR analysis showed that the expression of vimentin was increased in the following order: HCT-15 < Colo205 < HT29 < LoVo < SW480 (Fig. 1d, white bars). Next, we assessed the level of miR-17-5p in selected colon cancer cells via RT-qPCR (Fig. 1d). The level of miR-17-5p was relatively higher in CRC cell lines with a low vimentin expression compared with those with a high vimentin expression (Fig. 1d, black bars), indicating that the miR-17-5p level was inversely correlated with the metastatic potential of CRC cell lines.

### miR-17-5p regulates the expression of vimentin

**Fig. 2:**
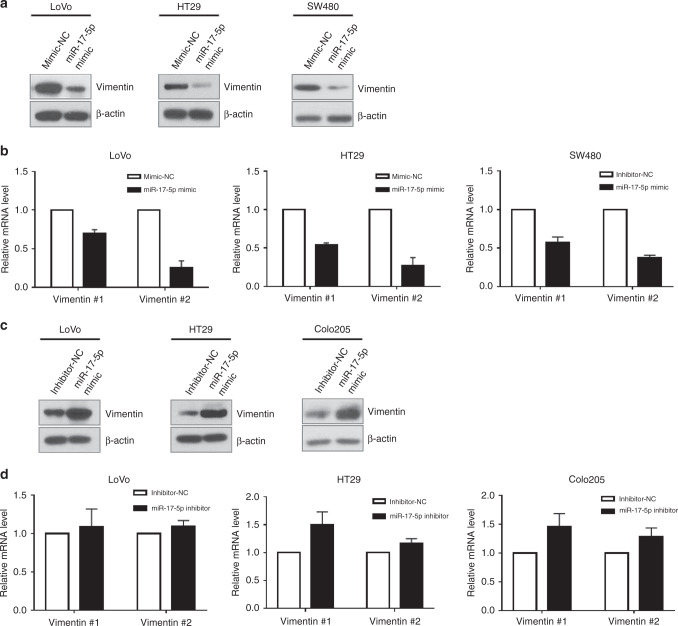
Regulation of vimentin expression by miR-17-5p in CRC cell lines. **a** Over-expression of miR-17-5p suppressed protein expression of vimentin in LoVo, HT29 and SW480. **b** Over-expression of miR-17-5p suppressed mRNA expression of vimentin in LoVo, HT29 and SW480. **c** Down-regulation of miR-17-5p increased the protein expression of vimentin, respectively. **d** Down-regulation of miR-17-5p did not increase the transcription of vimentin.

To determine if miR-17-5p regulated the expression of vimentin, colon cancer cells were transfected with miR-17-5p mimic or inhibitor for overexpression or inhibition, respectively. RT-qPCR analysis validated the increase or decrease in accordance to the level of miR-17-5p (Supplementary Fig. S2). Western blot analysis revealed that miR-17-5p mimic decreased the expression of vimentin in LoVo, HT29, and SW480 cells (Fig. 2a). Furthermore, *VIM* mRNA level was also reduced in miR-17-5p-overexpressed cells (Fig. 2b). In contrast, down-regulation of miR-17-5p by transducing miRNA inhibitor increased the expression of vimentin in LoVo, HT29, and Colo205 cells (Fig. 2c). The level of *VIM* mRNA was slightly increased via down-regulation of miR-17-5p (Fig. 2d). Based on these results, we speculated that miR-17-5p regulated the vimentin expression.

### miR-17-5p directly interacts with the 3′UTR of *VIM* mRNA

**Fig. 3:**
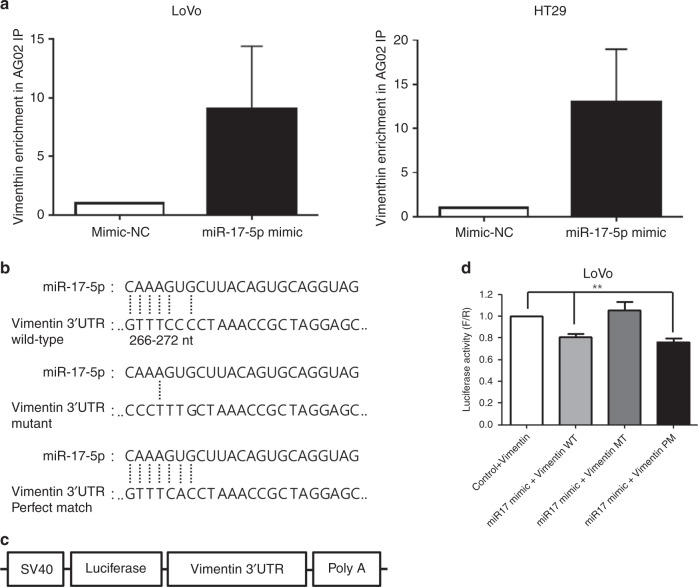
Direct targeting of miR-17-5p to the 3′UTR of *VIM* mRNA. **a** In LoVo and HT29 cell lines, the interaction between vimentin and miR-17-5p was assessed by RNP IP as described in the “Materials and methods” section. The expression of vimentin in the miR-17-5p IP materials was determined by RT-qPCR. **b** Schematic representation shows the construction of wild-type or mutant, or perfectly matched vimentin 3′UTR in the vector. **c** Schematic representation shows the plasmid containing the vimentin 3′UTR in luciferase assay. **d** Luciferase assays show a decrease in reporter activity after co-transfection with miRNA17 mimics and the wild-type vimentin 3′UTR, and the perfect match in LoVo cells. Mutant of vimentin 3′UTR did not show a decrease. The bars represent mean ± SD. **P < 0.001. All the experiments were repeated three times.

Based on the above results, we found that miR-17-5p suppresses vimentin expression. Therefore, we tested if miR-17-5p directly binds to the 3′UTR of *VIM* mRNA via Argonaute 2 (Ago2) ribonucleoprotein immunoprecipitation (RNP-IP). Ago2 RNP-IP revealed that *VIM* mRNA was more enriched in miR-17-5p-overexpressed LoVo and HT29 cells when compared with control miRNA, indicating that miR-17-5p directly binds to the 3′UTR of VIM mRNA. Representative results are shown in Fig. 3a and the results obtained from three independent experiments are presented in Supplementary Fig. S3. Direct interaction between miR-17-5p and the 3′UTR of *VIM* mRNA was subsequently assessed via luciferase-based assay. Interestingly, *VIM* mRNA is not predicted as a target mRNA of miR-17-5p in any prediction programs. However, we realised that only a single nucleotide of the 3′UTR of VIM mRNA was mismatched to the seed sequence of miR-17-5p (Fig. 3b). We constructed three luciferase reporter plasmids containing (1) wild-type 3′UTR of *VIM* mRNA harbouring a single nucleotide mismatch to the seed sequence of miR-17-5p (Fig. 3b, upper), (2) mutant type 3′UTR of *VIM* mRNA to block the binding of miR-17-5p (Fig. 3b, middle), and (3) mutant type 3′UTR of *VIM* mRNA which is perfectly matched to the seed sequence of miR-17-5p (Fig. 3b, lower). Each reporter was transduced into LoVo cells with control or miR-17-5p mimic, and the luciferase activity was determined. The luciferase reporter assay showed that overexpression of miR-17-5p suppressed luciferase expression in the wild-type and perfectly matched reporters. However, the luciferase expression was not influenced by mutant reporters (Fig. 3d). These results indicate that even a single nucleotide difference in the miRNA recognition element (MRE) led to target gene suppression by miR-17-5p. Furthermore, our findings suggest that target genes of miRNA are much more extensive than those predicted.

### miR-17-5p induces inhibition of cell migration and invasion

**Fig. 4:**
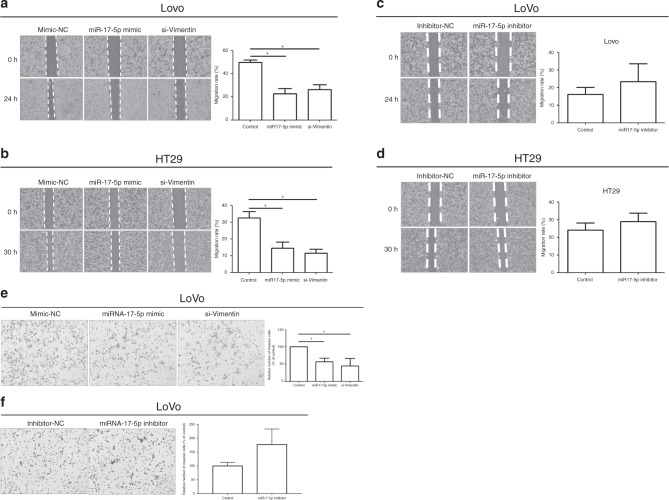
Control of cell migration and invasion by miR-17-5p. **a**, **b** Over-expression of miR-17-5p and silencing of vimentin decreased LoVo and HT29 cell mobility. Left: The width of the scratch-wounded cell monolayer was recorded at 0 and 24 h, or 0 and 30 h after wounding via photography. Right: The relative migration distance presented in the column chart. **c**, **d** Down-regulation of miR-17-5p increased cell migration in LoVo and HT29. **e** Over-expression of miR-17-5p and silencing of vimentin inhibited LoVo cell invasion based on Transwell assays. **f** Down-regulation of miR-17-5p increased cell invasion in LoVo. The bars represent mean ± SD. *P < 0.05. All the experiments were repeated three times.

To evaluate the role of miR-17-5p in controlling the metastatic potential of colon cancer, migratory and invasive activities were assessed by wound closure assay and Transwell invasion assay, respectively. In the wound closure assay, we found that miR-17-5p inhibited the migratory ability of LoVo and HT29 cells that was the same effect in *VIM*-silenced cells (Fig. 4a, b, respectively). Conversely, the decrease of miR-17-5p by transducing miRNA inhibitor enhanced the migratory ability of LoVo and HT29 cells (Fig. 4c, d, respectively). Invasiveness of colon cancer cells was also influenced by miR-17-5p. In LoVo cells, we observed that miR-17-5p inhibited invasive activity (Fig. 4e). Similarly, the number of invaded cells was decreased in *VIM*-silenced cells. However, the decreased expression of miR-17-5p showed higher invasiveness compared with control (Fig. 4f). These results demonstrate that miR-17-5p controlled the metastatic potential including migration and invasion of colon cancer cells by suppressing vimentin expression.

### miR-17-5p inhibits metastasis in mouse model

**Fig. 5:**
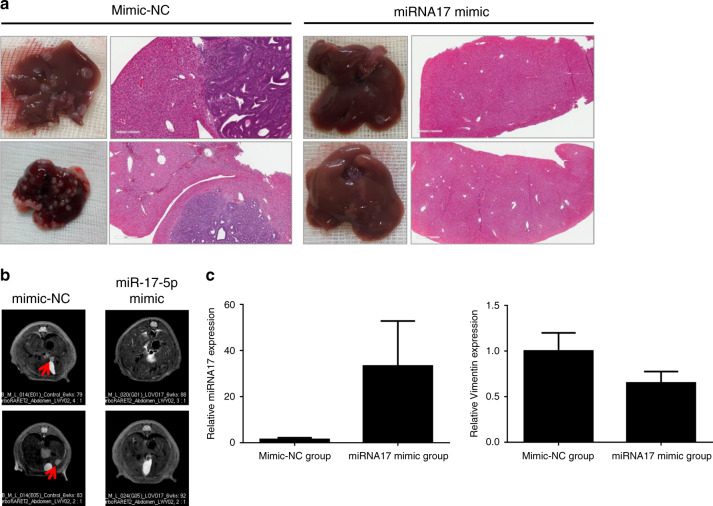
Inhibition of liver metastasis by miR-17-5p. **a** The H&E stain shows liver tissue of the control group (left); mice in the miRNA17 overexpressing group (right) are the same mice shown in Fig. 5b. **b** Magnetic resonance imaging (MRI) revealed liver metastasis (red arrows) in the metastatic mouse model with intra-splenic injection. **c** RT-PCR analysis revealed miRNA17 expression in the control group and the miRNA17 mimic group (left). RT-PCR analysis showed Vimentin expression in the control and miRNA17 mimic groups (right).

Based on the foregoing results, it is clear that miR-17-5p inhibits CRC metastasis in vitro. Additionally, to establish whether miR-17-5p activity was related to metastasis in vivo, we developed a metastatic mouse model by injecting miR-17-5p overexpressing LoVo cells. Seven BALB/c-nude mice were used in each group. Fewer metastases were found in the miR-17-5p overexpressing group injected with LoVo cells (14%, 1 out of 7 mice) than in the control group (71%, 5 mice out of 7). We collected metastatic tumour tissues and analysed the tissue histology to confirm tumour metastasis and detected the expression of miR-17-5p and Vimentin as a mesenchymal marker. Examination of metastatic tumours by H&E staining revealed liver metastatic nodules in the control group injected with LoVo cells (Fig. 5a, left panel), which were the same mice in Fig. 5b (left panel) but not in mice injected with miR-17-5p overexpressing LoVo cells (Fig. 5a, right panel) shown in Fig. 5b (right panel). MRI image revealed liver metastasis in control LoVo-injected mice during the monitoring period (Fig. 5b, left panel). However, no liver metastasis was detected on MRI in overexpressing miR-17-5p-injected LoVo cells (Fig. 5b, right panel). Increased expression of miR-17-5p compared with the control group was observed in the miR-17-5p overexpressing group (Fig. 5c, left panel). In addition, Vimentin expression was reduced in the miR-17-5p overexpressing group (Fig. 5c, right panel). Furthermore, to establish whether vimentin expression was related to metastasis in vivo, we developed a metastatic mouse model by injecting control LoVo cells and Vimentin-down-regulated LoVo cells (Supplementary Fig. S4A). Five BALB/c-nude mice were used in each group. Fewer metastases were found in the vimentin down-regulated group (0%, 0 mice out of 5) than the control group (80%, 4 mice out of 5) (Supplementary Fig. S4B). These data suggested that miR-17-5p reduced liver metastasis by targeting vimentin in vivo.

## Discussion

MiRNAs have been implicated in CRC tumorigenesis and metastasis, and represent effective molecular biomarkers for CRC diagnosis, prognosis, and therapy.^6,23^ In this study, we found that miR-17-5p expression was significantly down-regulated in primary CRC tissues with metastasis than in those without metastasis in our sequencing data, and its expression was inversely correlated with vimentin expression in CRC tissues and cell lines. In addition, we also found that miR-17-5p overexpression in CRC cell lines significantly decreased *VIM* mRNA expression, vimentin protein expression, cell migration, and invasion, whereas down-regulation of miR-17-5p in CRC cell lines increased vimentin protein expression, cell migration and invasion in vitro. Moreover, our findings suggested that overexpression of miR-17-5p inhibited liver metastasis in an intra-splenic injected mouse model. Additionally, our data indicated that miR-17-5p bound vimentin 3′UTR and regulated its expression. Therefore, our study indicated a new molecular biomarker for CRC prognosis.

Using RNP Ago2 IP and luciferase reporter assays, we identified vimentin 3′UTR as the new direct miR-17-5p downstream target. Although we established that miR-17-5p regulated vimentin expression, we found no specific target sequences in the prediction program, which only showed a perfect target match with miRNA seed. No perfect match was found between vimentin 3′UTR and miR-17-5p seed. We assumed a mismatch between miR-17-5p and vimentin based on Ago2 IP. The miRNAs exhibit six primary binding patterns defined according to the position of nucleotide binding between the miRNA and target mRNA.^24,25^ The seed mismatch^24^ occurs without adenosine in the binding position, and the imperfect centred match^25^ refers to the evolutionary conservation of the central region of miRNAs. Imperfect binding within the seed region can be resolved by a high degree of complementary binding at the 3′ end of the miRNA, facilitating the overall binding energetics of the miRNA-target.^24^ However, the seed matches are not currently shown by the binding prediction programs. Vimentin 3′UTR carries three binding sites of miR-17-5p with a single-point mismatch. The definition of seed region was disputed in the previously reported paper.^26,27^ The seed sequence is composed of at least six nucleotides beginning at the position two in core region.^27^ The core seed sequences include 6-mer (bases 2−7), 7-mer (“7-mer-A1” being bases 1−7, and “7-mer-m8” being bases 2−8), and 8-mers (bases 1−8).^27,28^ However, the prediction program did not show 7-mer-A1 and 8-mer. Therefore, we found the 6~8 mer with and without mismatch and evaluated the three binding sites via a luciferase assay. The first binding site, which was a 7-mer-A1 with 1 mismatch, reduced luciferase activity compared with the control. Although wild-type binding site was associated with partially reduced luciferase activity, the mutant site was restored. Additionally, a perfect match reduced the binding by a similar percentage compared with the wild type. Therefore, miR-17-5p bound to vimentin 3′UTR in spite of a binding mismatch.

In this study, we confirmed that miR-17-5p regulated EMT by binding vimentin 3′UTR in CRC. It has been reported that miR-17-5p is up-regulated in osteosarcoma^13^ and retinoblastoma,^14^ but acts as a metastatic inhibitor in non-small-cell lung cancer^15^ and breast cancer.^16^ Although miR-17-5p has varying function in each cancer type, it might be a metastatic suppressor or oncogene in an organ-dependent manner. Additionally, miR-17-5p has yielded controversial results in CRC.^17,18^ However, the reported studies and our results used different CRC cell lines and target genes. The binding site sequence of the target genes may differ due to point mutations in each cell line, and the binding force of miRNA may also vary. Because miRNAs exhibit different seed types and binding mismatch, they bind to different target genes in different cell lines and organs. MiR-17-5p is a representative miRNA with several binding types.^25^ Therefore, the differences in miR-17-5p expression and their contrasting effects in each cancer and CRC deserve further investigation.

Jiang et al.^18^ reported that up-regulated miR-17~92 acted as a negative regulator of activation in the Wnt/β-catenin pathway and inhibited CRC metastasis. The study showed that the overexpression of miR-17~92 reduced vimentin expression; however, the reduction of vimentin was regulated via inhibition of Wnt/β-catenin pathway by miR-17~92.^18^ In our data, miR-17-5p directly targeted vimentin 3′UTR and reduced vimentin expression. Therefore, miR-17-5p regulated EMT via inhibition of Wnt/β-catenin pathway as well as by directly targeting vimentin.

In conclusion, this study demonstrates that miR-17-5p regulates EMT by targeting vimentin in CRC. However, there are many targets of miR-17-5p other than vimentin. MiR-17-5p might regulate EMT by targeting other genes as well as vimentin. Six EMT-related genes, which are targeted by miR-17-5p, were found from Targetscan. The six genes are *STAT3*, *E2F1*, *HMGA2*, *SOX4*, *TWIST1*, and *EGFR* that have been studied to be associated with EMT of CRC.^5,29–38^ Although it has not been studied that the genes are regulated by miR-17-5p in CRC, miR-17-5p might regulate EMT by targeting these EMT-related genes. These findings provide novel insights into the molecular mechanisms of CRC metastasis. Therefore, this study might facilitate the development of new therapeutic strategies and novel biomarkers.

## Supplementary information

Supplementary figure

## Data Availability

The data supporting the finding of this study are available within the article and are available from the corresponding authors upon request.
