# Correction: MicroRNA-17-5p regulates EMT by targeting vimentin in colorectal cancer

**DOI:** 10.1038/s41416-020-1027-z

**Published:** 2020-08-20

**Authors:** Tae Won Kim, Yeo Song Lee, Nak Hyeon Yun, Chang Hoon Shin, Hye Kyung Hong, Hyeon Ho Kim, Yong Beom Cho

**Affiliations:** 1 Department of Health Sciences and Technology, SAIHST, Sungkyunkwan University, Seoul, Republic of Korea; 2 Sungkyunkwan University School of Medicine, Seoul, Republic of Korea; 3 Institute for Future Medicine, Samsung Medical Center, Seoul, Republic of Korea; 4 Department of Surgery, Samsung Medical Center, Sungkyunkwan University School of Medicine, Seoul, Republic of Korea

**Keywords:** Metastasis, Colorectal cancer

Correction to: *British Journal of Cancer* (2020); 10.1038/s41416-020-0940-5, published online 17 June 2020

Since the publication of this paper, the authors have noticed that Dr. Hyeon Ho Kim’s list of affiliations is not complete. The correct affiliations should include the Institute for Future Medicine, Samsung Medical Center, Seoul, Republic of Korea as well as the Department of Health Sciences and Technology, SAIHST, Sungkyunkwan University, Seoul, Republic of Korea. This error has been corrected above. The authors apologise for this oversight.

