# Supplementary material for: MicroRNA-17-5p regulates EMT by targeting vimentin in colorectal cancer
Source: Br J Cancer. 2020 Jun 17;123(7):1123–30. doi: 10.1038/s41416-020-0940-5 (PMC7524803; doi:10.1038/s41416-020-0940-5)
Supplement: Supplementary file 1 — Supplementary figure [file 41416_2020_940_MOESM1_ESM.docx]

**Supplementary Figure**

**
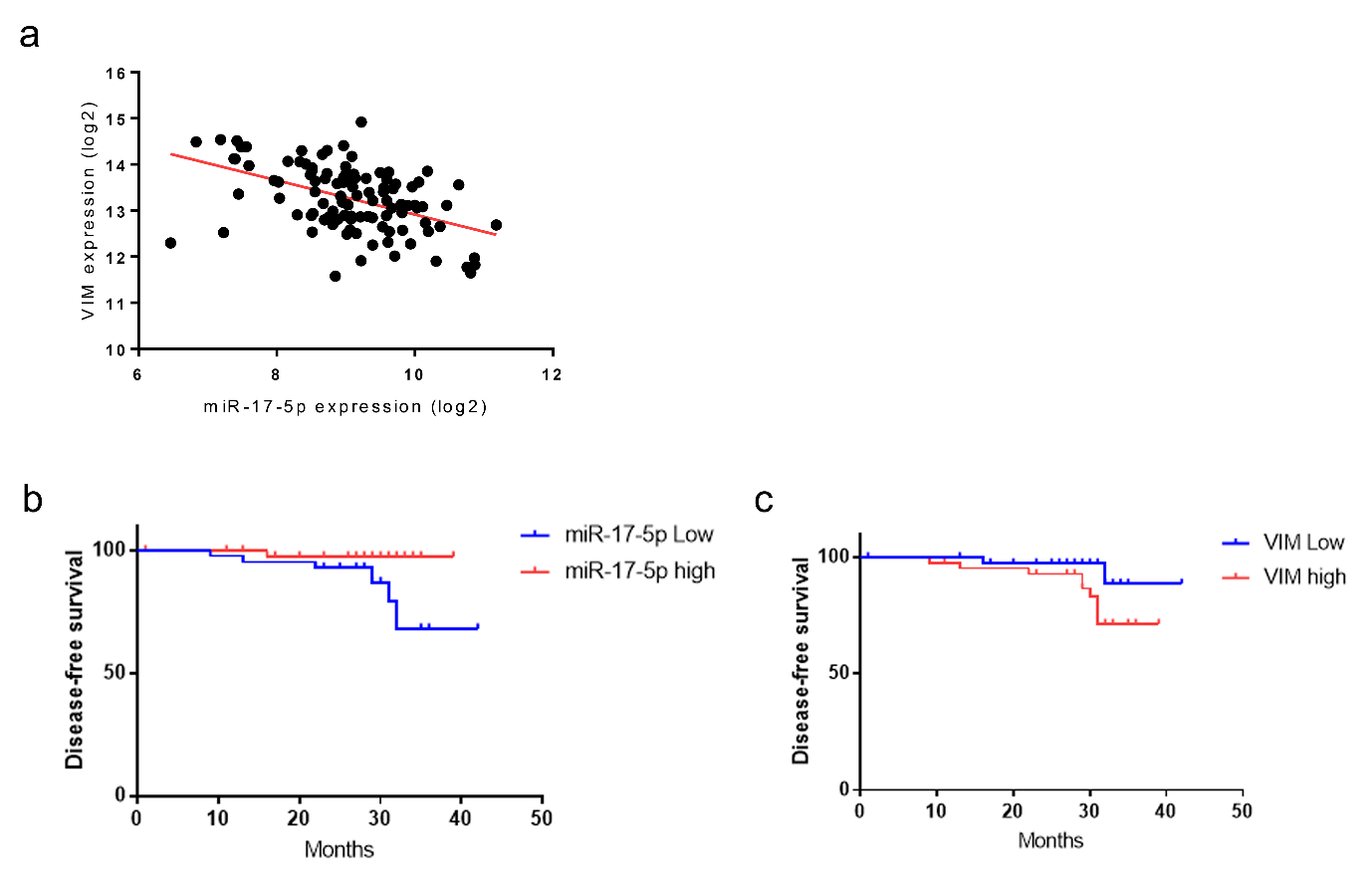
**

**Supplementary Fig. S1** Analysis from TCGA database. (a) Spearman correlation analysis showed a negative correlation between miR-17-5p and VIM expression (R=-0.4591, p<0.0001). (b) Disease-free survival analysis indicated that low level group of miR-17-5p showed poor prognosis compared to high level group (p=0.0189). (c) Disease-free survival analysis indicated that high level group of VIM expression showed poor prognosis than low level group (p=0.0386).


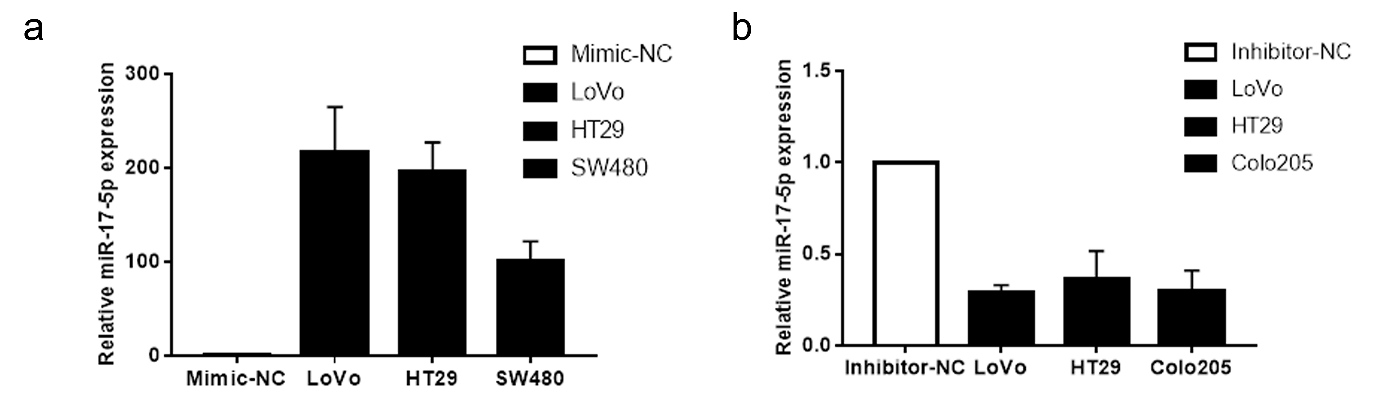


**Supplementary Fig. S2** Regulation of miR-17-5p by mimics or inhibitors of each CRC cell line. (a) The miR-17-5p level was increased by miR-17-5p mimic in each cell line. (b) The miR-17-5p level was decreased by miR-17-5p inhibitor in each cell line


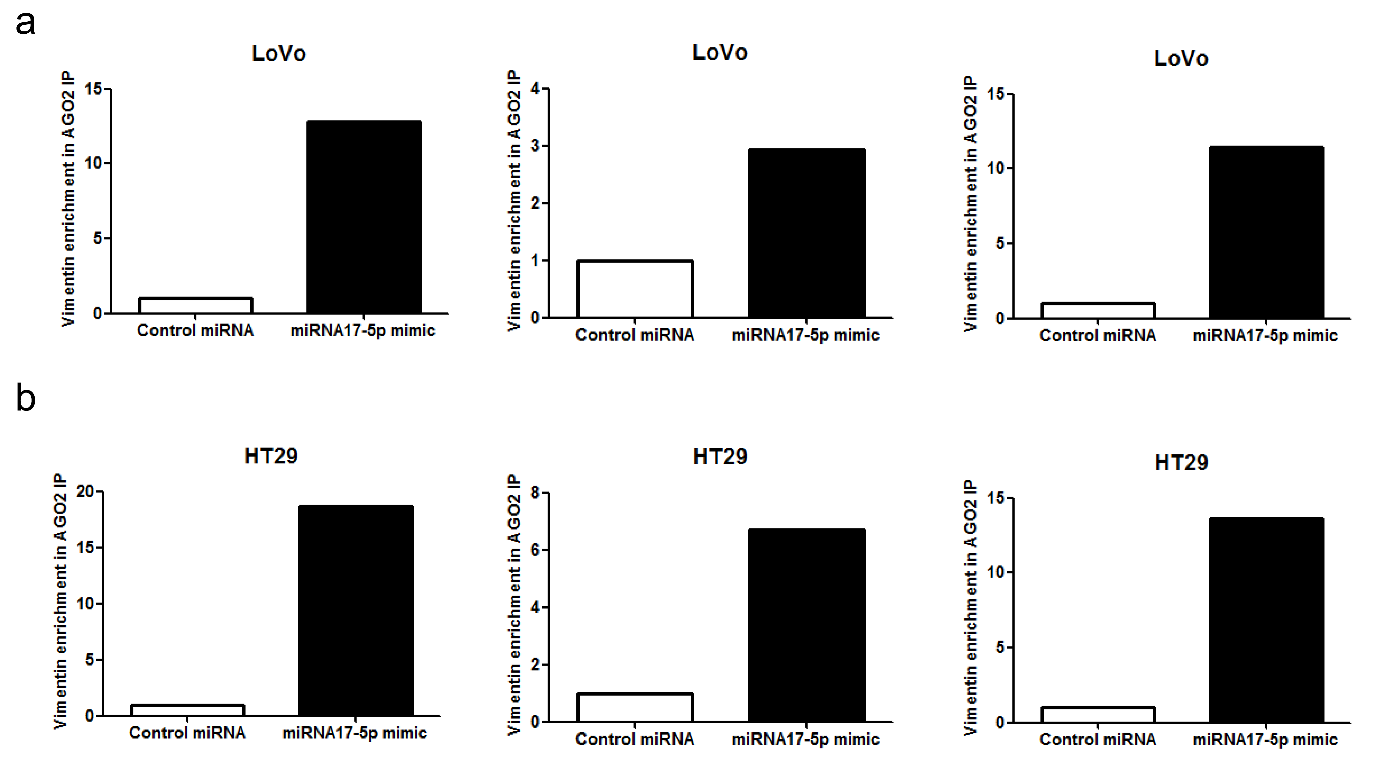


**Supplementary Fig. S3** Ago2 IP results in each experiment. (a) Interaction between vimentin and miR-17-5p was confirmed by Ago2 RNP IP repeated 3 times in LoVo; (b) Interaction between vimentin and miR-17-5p was confirmed by Ago2 RNP IP repeated 3 times in HT29.


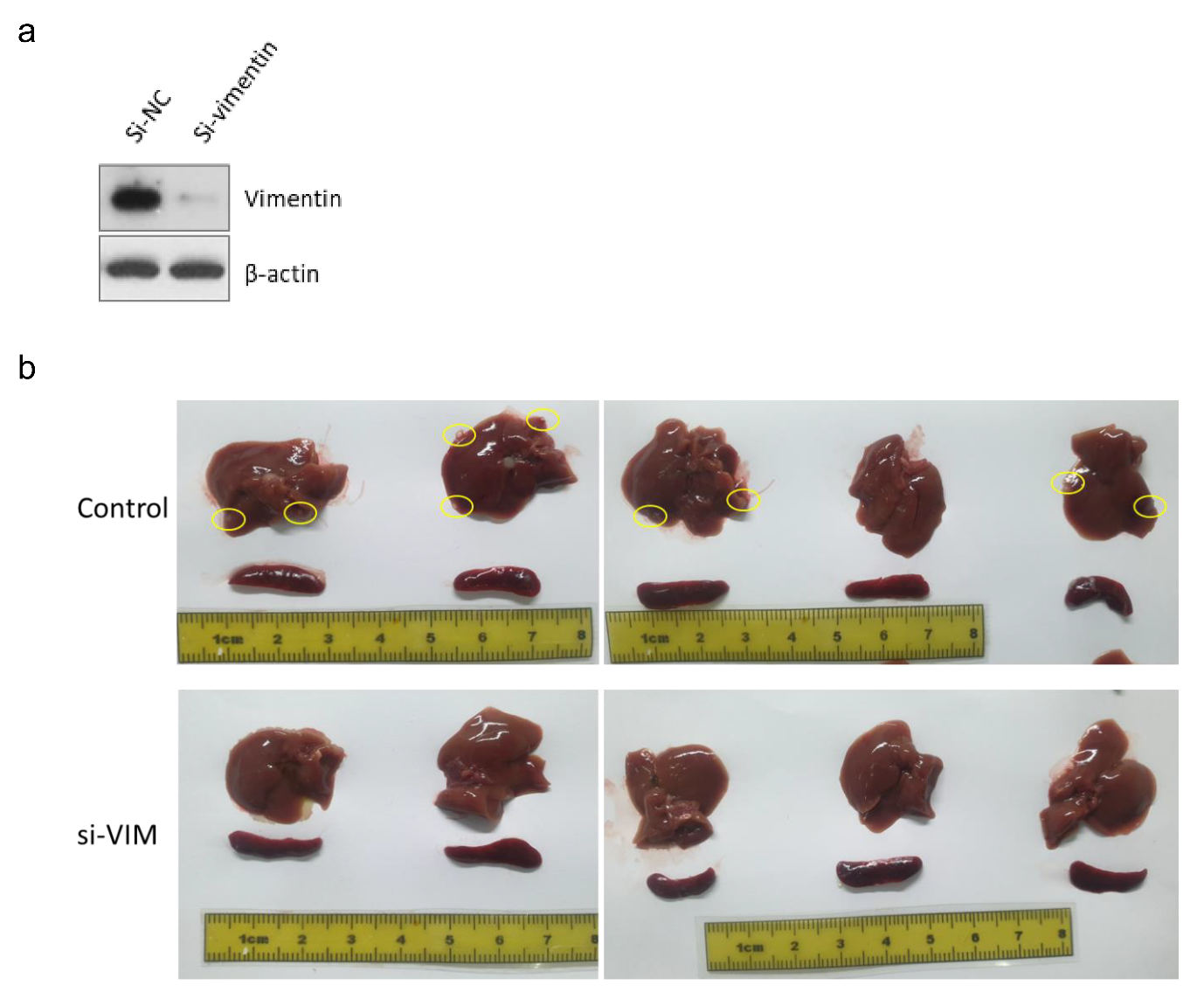


**Supplementary Fig. S4** Inhibition of liver metastasis by vimentin *in vivo*. (a)Western blot analysis revealed downregulation of vimentin by siRNA in LoVo cell. (b) Vmentin down-regulated group is fewer metastasis than control group.
